# Evaluation of the Distress Thermometer in Pelvic Exenteration: Responsiveness‐To‐Change, Convergent Validity, and Clinical Cutoff

**DOI:** 10.1002/pon.70565

**Published:** 2026-08-14

**Authors:** Nicholas Melander, Claudia Rutherford, Daniel Steffens, Judith Fethney, Sascha Karunaratne, Kate Alexander, Cherry Koh, Michael Solomon, Bora Kim

**Affiliations:** ^1^ School of Nursing and Midwifery The University of Notre Dame Australia Sydney NSW Australia; ^2^ Susan Wakil School of Nursing and Midwifery The University of Sydney Sydney NSW Australia; ^3^ The Daffodil Centre The University of Sydney, a Joint Venture With Cancer Council Sydney NSW Australia; ^4^ Cancer Care Research Unit Sydney Local Health District NSW Australia; ^5^ Surgical Outcomes Research Centre (SOuRCe) Royal Prince Alfred Hospital Sydney NSW Australia; ^6^ Faculty of Medicine and Health Central Clinical School The University of Sydney Sydney NSW Australia; ^7^ Sydney Clinical Trials Centre Faculty of Medicine and Health, The University of Sydney NSW Australia

**Keywords:** cancer, distress thermometer, oncology, pelvic exenteration, pelvic neoplasms, psychological distress

## Abstract

**Background:**

Pelvic exenteration (PE) is a radical surgery for advanced pelvic malignancies that carries substantial psychosocial impacts. Assessing distress is critical for timely support.

**Aims:**

To evaluate the responsiveness‐to‐change and convergent validity of the distress thermometer (DT), and to determine an optimal cutoff score for detecting clinically significant distress in a PE cohort.

**Methods:**

Secondary analyses of cohort study data involving adult patients who underwent PE at a quaternary referral hospital. Responsiveness was evaluated by determining whether the DT differentiated cases of decreased emotional well‐being and health‐related quality of life from pre‐ to post‐surgery, using the Functional Assessment of Cancer Therapy‐General (FACT‐G), FACT‐Colorectal, and Emotional Well‐Being (EWB) subscale as external criteria. Convergent validity was evaluated using the correlation between the DT and the EWB subscale scores. Cutoff scores were examined by reviewing the following indices, using an existing EWB cutoff as an external anchor: sensitivity, specificity, positive/negative predictive values, and clinical utility index (CUI).

**Results:**

The analysis included 377 patients (mean age: 61 years, 56% male; 41% recurrent rectal cancer). The DT demonstrated responsiveness, with AUCs > 0.7 for detecting emotional and quality‐of‐life decline post‐surgery. Convergent validity was demonstrated through a moderate correlation between the DT and the EWB subscale scores (*r*_s_ = −0.56, *p* < 0.001). The optimal DT cutoff score was 4: Sensitivity; 87.0%, Specificity; 52.7%, CUI− value; 0.49 (ruling out cases: satisfactory utility), CUI+ value; 0.33 (case finding: poor utility).

**Conclusion:**

The DT is a valid and responsive tool for detecting psychological distress in PE patients. Its high sensitivity supports its use in routine screening.

## 1 Background

Pelvic exenteration (PE) is a radical surgical procedure involving the partial or complete removal of organs in the pelvic area. It is often the only curative treatment option for selected patients with recurrent or locally invasive pelvic malignancies, including colorectal, gynaecological, and urological cancers [1]. The invasive and extensive nature of this surgery impacts multiple health‐related quality of life (HRQL) domains, with previous research highlighting significant physical, psychological and social impacts experienced after treatment completion [2]. Qualitative findings further describe patients' feelings of helplessness, frustration, and anger, as well as feeling unprepared and uncertain about the impact that the surgery may have on their daily lives [3]. Such challenges place these patients at an increased risk of psychological morbidity [4].

The most widely used definition of distress is offered by the National Comprehensive Cancer Network (NCCN) [5, p. 5], who describe distress as a “*multifactorial unpleasant experience of a psychological (*i.e.*, cognitive, behavioural, emotional), social, spiritual, and/or physical nature that may interfere with one's ability to cope effectively with cancer, its physical symptoms, and its treatment*”. They go on to suggest that distress exists on a spectrum and can vary from common feelings of sadness and fear, to more severe and disabling problems such as depression, anxiety and panic. The presence of psychological distress has been associated with negative health outcomes in cancer patients, including a reduced HRQL [6] and increased physical symptom burden [7, 8].

The timely identification and management of psychological distress is increasingly considered an imperative of quality cancer care [9, 10], with the NCCN recommending that patients be screened for distress regularly throughout their illness. Despite these recommendations, screening rates remain as low as 40% in reported literature [11], with previously used screening tools having been criticized as being lengthy and bothersome [12]. As a result, the Distress Thermometer (DT) has been proposed and utilized as an ultra‐short and easy to use screening tool that is effective at identifying clinically significant distress, whilst remaining minimally disruptive to clinical practice [9]. Developed by Roth et al. [13], the DT is a single‐item visual scale, in which respondents rate their distress from 0 to 10, with 10 indicating extreme distress.

The DT has evidence of validity for identifying distress in patients with various solid cancer types, including colorectal [14] and gynecological cancer [15]. However, despite the high psychological morbidity and complex care needs, there are currently no studies that have evaluated the DT amongst patients who have undergone major oncologic surgeries such as pelvic exenteration. Additionally, the optimal cutoff score for the DT varies depending on the clinical setting and cancer types [16], highlighting the need to determine the appropriate cutoff specifically for this surgical cohort.

The primary aim of this study was to examine the responsiveness‐to‐change (hereby referred to as responsiveness) and convergent validity of the DT in patients before pelvic exenteration and during follow‐up. The secondary aim was to assess the accuracy of identifying individuals with psychological distress and to examine clinically meaningful cutoff points for identifying this patient group in this surgical cohort.

## 2 Methods

### 2.1 Study Design

This study is a secondary analysis of a prospective cohort study conducted at a major quaternary referral hospital in Sydney, Australia, which examines clinical and HRQL surgical outcomes of pelvic exenteration on an ongoing basis [17]. The study received ethical approval and governance authorization from the Royal Prince Alfred Hospital Human Research Ethics and Governance Office (X13‐0283 and X20‐0392 for the original conduct of the study, and X24‐0115 for secondary analysis).

### 2.2 Participants and Consent Procedures

The detailed recruitment process for the original study is described elsewhere [17]. In brief, consecutive adult patients aged 18 or above who were scheduled to receive pelvic exenteration to treat advanced primary or recurrent malignancies within the pelvis were invited to participate. Interested patients provided informed consent after being briefed on the study and procedures by an experienced research officer.

### 2.3 Data Sources/Collection

In the original cohort study, participants' baseline clinical and sociodemographic data were collected by a research officer and an experienced pelvic exenteration clinical fellow via electronic medical records (Table 1). For the patient‐reported outcomes, participants were asked to complete a questionnaire at baseline (prior to receiving surgery) and post‐operatively up to 60 months. Patient self‐reported questionnaire responses were collected via mail, online survey, or through telephone‐assisted completion as per the participants' preference, with two reminders for non‐respondents. Those who did not respond after the reminders were recorded as lost to follow‐up.

**TABLE 1:** Baseline characteristics of study participants.

	Participants included in the analysis (Total *n* = 377)		Participants not included in the analysis (Total *n* = 771)		*p* value^a^
	*N*	%	*N*	%	
Demographic characteristics					
Age, mean (SD)	61.2 (11.5)		59.0 (13.4)		0.005
Sex					0.166
Female	165	43.8	372	48.2	
Male	212	56.2	399	51.8	
Marital status					0.065
Divorced	35	9.3	57	7.4	
Single	52	13.8	93	12.1	
Widowed	18	4.8	27	3.5	
Married/living with partner	271	71.9	309	40.1	
Missing	1	0.3	285	37.0	
Location					0.014
NSW Regional/Rural	147	39.0	285	37.0	
Interstate	78	20.7	127	16.5	
Overseas	0	0	14	1.8	
NSW Metro	152	40.3	342	44.4	
Missing	0	0	3	0.4	
Education					0.438
None/Primary school only	12	3.2	14	1.8	
Year 10–12	167	44.3	136	17.6	
TAFE certificate or diploma	77	20.4	63	8.2	
University degree	105	27.9	68	8.8	
Missing	16	4.2	490	63.6	
Employment					0.058
Part/Full time	146	38.7	128	16.6	
Home duties	28	7.4	13	1.7	
Unemployed	37	9.8	43	5.6	
Retired	154	40.8	106	13.7	
Missing	12	3.2	481	62.4	
Clinical characteristics					
Cancer type					< 0.001
Non‐cancer	10	2.7	28	3.6	
Primary other^b^	50	13.3	129	16.7	
Primary rectal	95	25.2	232	30.1	
Recurrent other^b^	66	17.5	175	22.7	
Recurrent rectal	156	41.4	202	26.2	
Treatment intent					< 0.001
Palliative	9	2.4	58	7.5	
Curative	368	97.6	706	91.6	
Missing	0	0	7	0.9	
Neoadjuvant radiotherapy					0.275
No	200	53.1	419	54.3	
Yes	166	44.0	302	39.2	
Missing	11	2.9	50	6.5	
Neoadjuvant chemotherapy					0.089
No	182	48.3	406	52.7	
Yes	185	49.1	332	43.1	
Missing	10	2.7	33	4.3	
Surgical margin					0.670
R0	283	75.1	556	72.1	
R1/R2	83	22.0	174	22.6	
Missing	11	2.9	41	5.3	
Exenteration type					0.011
Partial	181	48.0	429	55.6	
Complete	196	52.0	337	43.7	
Missing	0	0	5	0.6	
Stoma					0.660
No	115	30.5	225	29.2	
Yes	262	69.5	545	70.7	

^a^ *p* values for study participants who were included versus those who were not included in the analysis using *t*‐tests (for continuous variables) or Pearson Chi‐Square tests (for categorical variables).

^b^ Includes colon, rectosigmoid, sigmoid, gynecological, genitourinary, sarcoma/soft tissue, anal, melanoma.

The current validation study includes patient‐reported outcomes data collected at both baseline and at the 6‐month post‐surgical follow‐up between 2008 and April 2024. Specifically, we included patient cases with DT scores and scores for at least one other patient‐reported outcome measure that allowed one or more planned analyses (Table 2).

**TABLE 2:** Baseline, 6‐month follow‐up, and mean changes in DT, FACT‐G, FACT‐C, and EWB subscale scores.

	Baseline		6 month follow‐up				
Measures/subscale	*N*	Mean (SD)	*N*	Mean (SD)	Mean change (95% CI)^e^	*N*	*p* value
DT^a^ ^,^ ^d^	377	4.29 (3.02)	377	2.88 (2.74)	−1.41 (−1.76, −1.07)	377	< 0.001
FACT‐G^b^ ^,^ ^d^	367	75.74 (17.59)	368	77.63 (17.65)	1.99 (0.16, 3.82)	358	0.033
FACT‐C^b^ ^,^ ^d^	368	94.90 (21.33)	369	96.92 (21.05)	2.06 (−0.15, 4.28)	360	0.068
EWB^c^ ^,^ ^d^	371	16.83 (5.01)	371	18.50 (4.7)	1.63 (1.16, 2.09)	365	< 0.001

Abbreviations: DT, distress thermometer; FACT‐G, The Functional Assessment of Cancer Therapy‐General; FACT‐C, Functional Assessment of Cancer Therapy – Colorectal; SD, standard deviation.

^a^ Higher scores indicate a higher level of distress.

^b^ Higher scores indicate better health‐related quality of life.

^c^ A subscale of FACT‐G and FACT‐C. Higher scores indicate better emotional well‐being.

^d^ Maximum and minimum scores of the measures: DT (0–10), FACT‐G (0–108), FACT‐C (0–136), EWB (0–24).

^e^ Comparison between baseline and 6‐month follow‐up using paired sample *t* test (Significant with *p* < 0.05).

### 2.4 Measures

The DT is a single‐item visual analogue scale that measures respondents' self‐reported psychological distress level over the past week. It has an image of a thermometer with a scale ranging from 0 (no distress) to 10 (extreme distress) [13].

The Functional Assessment of Cancer Therapy‐General (FACT‐G) [18] is a tool used to assess the HRQL of patients with cancer and is one of the most widely used measures in cancer clinical trials [19]. It consists of 27 items with four subscales: physical well‐being (PWB), social/family well‐being (SFWB), emotional well‐being (EWB), and functional well‐being (FWB). Responses to each FACT‐G item range from 0 (not at all) to 4 (very much), with a 7‐day recall period. FACT‐G generates summative scores ranging between 0 and 108, with a higher score indicating better HRQL. The EWB subscale comprises six items, with the summative score ranging between 0 and 24 and a higher score indicating better emotional well‐being. Both FACT‐G and the EWB subscale were used in this study.

The Functional Assessment of Cancer Therapy‐Colorectal (FACT‐C) [20] assesses HRQL for patients with colorectal cancer. It comprises the four HRQL subscales contained in FACT‐G, with an additional subscale assessing colorectal cancer specific concerns (9 items). The summative score of FACT‐C is the sum of all five subscales, with a total score ranging from 0 to 136. A higher score indicates better HRQL. The reliability [21, 22, 23], discriminant validity [21, 23], convergent validity [21, 22, 23], and responsiveness to change [22, 23] of the FACT‐G and FACT‐C have been extensively evaluated across different cancer types.

### 2.5 Statistical Analysis

IBM SPSS version 29 was used for statistical analyses. Descriptive statistics were used to summarize the clinical and sociodemographic characteristics of the study participants, as well as their DT scores, and summative FACT‐G and FACT‐C scores. These were reported in frequencies, percentages, mean, and standard deviation (SD) and median as appropriate. Only available data were used, without data imputation. For all statistical tests, *p* values < 0.05 were considered statistically significant.

### 2.6 Convergent Validity

Convergent validity was examined by assessing distress and a related construct, emotional well‐being, using the EWB subscale [24]. We hypothesized moderate correlations using Spearman's rank correlation coefficient, with an expected absolute rho value of 0.30–0.59. Moderate correlations were hypothesized as the EWB subscale was a construct related to distress, but not exactly the same.

#### 2.6.1 Responsiveness to Change

External responsiveness is defined as “*the extent to which changes in a measure over a specified time frame are related to corresponding changes in a reference measure of health status*” [25, p. 459]. External responsiveness was assessed by examining whether the DT scores showed the same directional changes observed in reference measures—in this case, the HRQL indicated by the summative FACT‐G and FACT‐C scores and emotional well‐being indicated by the EWB subscale.

We used the Minimal Important Difference (MID) previously reported for FACT‐G (5–6 points) [26], FACT‐C (5–8 points) [27], and EWB (2–3 points) [27]. Using the upper and lower bounds of FACT‐G and FACT‐C MIDs, we categorized the sample into two groups: individuals with HRQL decline from baseline to 6 months post PE surgery versus those without HRQL decline. Similarly, using the upper and lower bounds of EWB MID, we categorized the sample into the following groups: those with a decline in emotional well‐being from baseline to 6 months versus those without emotional well‐being decline.

The Receiver Operating Characteristic (ROC) and the area under the ROC (AUC) analysis was conducted to assess external responsiveness; specifically, whether changes in DT scores from baseline to 6 months post‐surgery could discriminate between the above mentioned binary groups with the same directional change, with an AUC of ≥ 0.7 with 95% confidence intervals considered adequate for discriminating between groups [28]. Additionally, Pearson correlation analysis was used to assess external responsiveness by examining the strength of the correlation between the DT score changes and the reference measure score changes (FACT‐G, FACT‐C, and EWB) from baseline to 6 months.

### 2.7 Examining Optimal Cutoff Points

To assess the accuracy of the DT and the optimal cutoff point for identifying individuals with clinically significant distress, we used the existing cutoff scores of the summative EWB subscale as an anchor. A correlation analysis was first conducted between EWB and DT scores to assess the suitability of the EWB scale as an anchor, with a correlation coefficient of ≥ 0.5 considered indicative of an appropriate anchor. After confirming suitability, the EWB cutoff score of ≤ 13 (for poor emotional well‐being) [29] was used to create two groups: those with poor emotional well‐being and those with fair to good emotional well‐being. ROC analysis was used to determine whether the DT adequately distinguished between these groups at both baseline and 6 months by examining the AUC, with values of ≥ 0.7 considered adequate for its discrimination [28].

Optimal cutoffs were determined using ROC analysis by assessing the following indices, at both baseline and 6 months: sensitivity, specificity, positive predictive value (PPV) and negative predictive value (NPV) [30]. Additionally, positive Clinical Utility Index (CUI+) and negative Clinical Utility Index (CUI−) were examined. CUI provides useful indices to examine the clinical usefulness of a diagnostic test by assessing its discrimination capacity while accounting for the prevalence of positive or negative cases [31]. The CUI+ was calculated by multiplying sensitivity and PPV, which indicates its degree of rule‐in accuracy (i.e., case finding). The CUI− was calculated by multiplying specificity and NPV to assess rule‐out accuracy [31]. CUIs were then classified as follows: excellent utility ≥ 0.81, good utility ≥ 0.64, satisfactory utility ≥ 0.49, and poor utility < 0.49 [31, 32].

## 3 Results

### 3.1 Sample Characteristics

In the original study, 1148 participants were enrolled. Our secondary analysis included 377 patients who met the eligibility criteria. Baseline sociodemographic and clinical characteristics of the patients are shown in Table 1. Participants had a mean age of 61.2 years and 56.2% were male. The most common cancer type was recurrent rectal cancer (41.4%) and the majority received curative treatment (97.6%).

### 3.2 Distress and HRQL Scores

The mean DT score was 4.29 (SD 3.02) at baseline, and this decreased to 2.88 (SD 2.74) at 6 months. Table 2 provides the mean scores for the patient‐reported outcome measures used in the analysis. Supplementary 1 provides the frequency distribution of DT scores.

### 3.3 Convergent Validity

There was a moderate correlation between the DT score and the EWB score at baseline (*r*_s_ = −0.56, *p* < 0.001, *n* = 371) and at 6 months (*r*_s_ = −0.57, *p* < 0.001, *n* = 371) as hypothesized, demonstrating convergent validity of the DT with this subscale.

### 3.4 External Responsiveness

All AUCs exceeded our prespecified criterion of 0.7, suggesting that the change in DT scores adequately distinguished between patients with and without clinically meaningful declines in HRQL, as indicated by FACT‐G and FACT‐C scores (Table 3). In other words, when FACT‐G and FACT‐C scores showed a clinically meaningful decline in HRQL (reductions meeting the specified MIDs), DT scores also increased, indicating worsening distress. When the FACT‐G score decreased by five or more points from baseline to 6 months post‐surgery, the mean DT score increased by 0.69. In the group whose FACT‐G score did not show a clinically meaningful decline in HRQL, the mean DT score decreased by 2.42 over the same period (Table 3).

**TABLE 3:** Mean difference in DT scores and AUCs for discriminating between patients with and without clinically meaningful declines in HRQL or emotional well‐being.

Measures and MID used to categorize groups	Groups^a^	Mean difference in DT score (6 months minus baseline)	AUC^b^ (95% CI)
FACT‐G, MID 5^c^	Clinically meaningful HRQL decline	0.69	0.76 (0.71–0.81)
	No decline in HRQL	−2.42	
FACT‐G, MID 6^c^	Clinically meaningful HRQL decline	0.67	0.75 (0.70–0.81)
	No decline in HRQL	−2.40	
FACT‐C, MID 5^d^	Clinically meaningful HRQL decline	0.48	0.75 (0.69–0.80)
	No decline in HRQL	−2.47	
FACT‐C, MID 8^d^	Clinically meaningful HRQL decline	0.67	0.74 (0.69–0.80)
	No decline in HRQL	−2.35	
EWB^e^, MID 2^e^	Clinically meaningful EWB decline	0.68	0.72 (0.66–0.79)
	No decline in EWB	−2.05	
EWB^e^, MID 3^e^	Clinically meaningful EWB decline	1.19	0.74 (0.67–0.82)
	No decline in EWB	−1.92	

Abbreviations: AUC: Area under the ROC (receiver operating characteristic) curve; DT: distress thermometer; FACT‐C: Functional Assessment of Cancer Therapy – FACT‐G: The Functional Assessment of Cancer Therapy‐General; Colorectal; HRQL: health‐related quality of life; MID: Minimal Important Difference.

^a^ Groups were categorized into clinically meaningful decline in HRQL or emotional well‐being versus non‐decline (including stable and improved individuals).

^b^ An AUC ≥ 0.7 was considered adequate for distinguishing between the groups.

^c^ The total sample included in the analysis was 358, excluding those with missing FACT‐G data.

^d^ The total sample included in the analysis was 360, excluding those with missing FACT‐C data.

^e^ The total sample included in the analysis was 365, excluding those with missing EWB subscale data.

Similar results were found when using the EWB subscale, where the DT distinguished between the binary groups: those with a clinically meaningful decline in emotional well‐being versus those without such decline, as indicated by our prespecified AUC of above 0.7 for confirming responsiveness. The mean DT score differences between groups and their respective AUCs are detailed in Table 3.

DT and three external criteria also demonstrated a consistent directional change from baseline to 6 months using Pearson correlation analysis: as DT scores increased, FACT‐G, FACT‐C and EWB scores decreased, with moderate correlation coefficients between DT and all three measures (FACT‐G: *r* = −0.55, *p* < 0.001; FACT‐C: *r* = −0.54, *p* < 0.001; EWB: *r* = −0.47, *p* < 0.001).

### 3.5 Exploration of the DT Cutoff

A strong correlation between EWB and DT at both baseline (*r* = −0.55, *p* < 0.001) and 6 months post‐surgery (*r* = −0.58, *p* < 0.001) demonstrated the suitability of EWB as an anchor for identifying a clinically meaningful cutoff for DT. Poor emotional well‐being was reported by 24.4% (*n* = 92) of the sample at baseline and 15.9% (*n* = 60) at 6 months, as indicated by the previously reported EWB cutoff score [29]. The AUC for distinguishing the poor EWB group was 0.78 (95% CI; 0.73–0.83) at baseline, suggesting fair discrimination [28], and it increased to 0.81 (95% CI; 0.75–0.87) at 6 months, indicating good discrimination [28] (Supplementary 2). The frequency distribution graphs of the DT scores, grouped by the EWB cutoff at baseline and 6 months, are presented in Supplementary 3.

Sensitivity, specificity, PPV and NPV for binary groups within the distribution of DT scores at baseline and 6 months are presented in Table 4. The measures presented in Table 4 using baseline data support an optimal cutoff DT score of 4 against EWB, when high sensitivity was prioritized to increase confidence in identifying more true positive cases, with sensitivity of 87.0% (80/92) and specificity of 52.7% (147/279). The CUI+ value was 0.33, indicating poor utility for ruling in cases, while the CUI− value of 0.49 indicates satisfactory utility for ruling out cases.

**TABLE 4:** Measures of accuracy for each DT cutoff score at baseline and at 6 months.

DT cutoff Score	EWB (poor emotional well‐being)^a^ Baseline						At 6 months					
	Sensitivity (%)	Specificity (%)	PPV (%)	NPV (%)	CUI+	CUI−	Sensitivity (%)	Specificity (%)	PPV (%)	NPV (%)	CUI+	CUI−
1	98.9	16.8	28.2	97.9	0.28	0.16	95.0	30.2	20.8	96.9	0.20	0.29
2	95.7	32.3	31.8	95.7	0.30	0.31	91.7	45.7	24.6	96.6	0.23	0.44
3	90.2	43.0	34.3	93.0	0.31	0.40	80.0	63.3	29.6	94.3	0.24	0.60
4	87.0	52.7	37.7	92.5	0.33	0.49	73.3	72.7	34.1	93.4	0.25	0.68
5	81.5	58.4	39.3	90.6	0.32	0.53	66.7	80.7	40.0	92.6	0.27	0.75
6	69.6	73.5	46.4	88.0	0.32	0.65	55.0	90.7	53.2	91.3	0.29	0.83
7	54.3	82.4	50.5	84.6	0.27	0.70	41.7	92.9	53.2	89.2	0.22	0.83
8	43.5	90.7	60.6	83.0	0.26	0.75	25.0	96.5	57.7	87.0	0.14	0.84
9	18.5	94.6	53.1	77.9	0.10	0.74	15.0	99.0	75.0	85.8	0.11	0.85
10	13.0	97.1	60.0	77.2	0.08	0.75	10.0	99.7	85.7	85.2	0.09	0.85

Abbreviations: CUI+, positive clinical utility index; CUI−, negative clinical utility index; DT, distress thermometer; EWB, emotional well‐being subscale; NPV, negative predictive value; PPV, positive predictive value.

^a^ The total sample included in the analysis was 371, excluding those with missing EWB data at baseline or at 6 months.

Table 4 presents measures of accuracy for each DT cutoff score at baseline and at 6 months. Overall, 4 was the optimal cutoff against EWB, with reduced sensitivity (73.3%, 44/60) and improved specificity (72.7%, 226/311) compared to the baseline proposed cutoff of 4. Prioritizing high specificity over sensitivity to reduce false positivity, a cutoff of 5 could also be considered with good utility for ruling out cases (CUI− of 0.75), but CUI+ remained at poor utility for case finding (0.27).

## 4 Discussion

This study provides the first validation of the DT in patients receiving pelvic exenteration. The DT demonstrated satisfactory convergent validity, showing a moderate correlation with the EWB subscale, consistent with prior research supporting the DT's convergent validity [33, 34]. Our study also found that the DT was responsive to changes across three external criterion measures from baseline to 6 months post‐surgery. The mean increase in DT scores in groups with a decline in HRQL and emotional well‐being suggests a consistent directional change as per the external criterion.

Two prior studies have evaluated the DT's responsiveness. Over a decade ago, Gessler et al. [35] demonstrated low to moderate responsiveness in a sample of patients with various cancer types attending outpatient clinics, yet despite calling for further research, evidence remains limited. Only Thalen‐Lindstrom et al. [36] advanced these findings, reporting responsiveness of the DT in oncology patients. However, both studies involved heterogeneous patient cohorts regardless of treatment status or cancer type, limiting the generalizability of their findings to a specific clinical cohort to inform clinical practice. Finding that the DT was responsive pre and post‐surgery, our study builds on the limited existing evidence and provides additional support for its applicability in longer‐term monitoring of distress, particularly in patients undergoing oncologic surgeries. Given the reported gaps and unmet psychological and emotional needs in post‐treatment care for cancer survivors [37, 38, 39], this finding is particularly noteworthy.

Our proposed optimal cutoff score of 4 demonstrated acceptable to good discrimination against emotional well‐being as an anchor, with AUC values of 0.78 and 0.81 at the respective time points. From a cutoff score of 4, the DT began to demonstrate fair to good clinical utility for ruling out cases at baseline and 6 months, respectively, with good sensitivity, compared to a cutoff of 3, which showed poor utility at both time points. This cutoff is consistent with current NCCN guidelines, which suggest a cutoff of 4 [5, p. 5], and is further supported by two meta‐analyses of mixed cancer cohorts across varying clinical stages [16, 40], as well as in a study in a cancer survivorship cohort [41]. Our findings are also consistent with the original 2005 study by Jacobsen et al. [42], which first established this cutoff score to distinguish clinically significant distress. Collectively, these results, and others like them, reinforce the broad clinical applicability of this cutoff score across cancer populations and settings, including post‐oncologic surgeries as demonstrated in this study. Whilst a cutoff of 5 has been suggested in other survivorship studies [33], we found that ≥ 4 provided a higher sensitivity than ≥ 5 and that increasing the optimal cutoff to ≥ 5 did not result in a better clinical utility index.

Previous literature has argued for prioritizing sensitivity over specificity when determining an optimal cutoff for screening tools [43] such as the DT to ensure that possible cases of psychological morbidity were not missed. This is particularly relevant in the post‐treatment and survivorship context, where patients may have less frequent contact with healthcare professionals and fewer opportunities for identifying psychological distress. In pelvic exenteration patients, this concern is compounded by the extensive nature of their surgery, impacting a wide range of HRQL domains [3]. This also aligns with other studies involving cancer survivors, where prioritizing sensitivity over specificity in screening tools has been emphasized to address the risk of undetected distress [33, 37, 38].

### 4.1 Clinical Implications

Low reported specificity in previous literature (53%–62% [33, 43, 44]) and in our study (53% at baseline) suggests that the DT should be used solely as a screening tool and may not be appropriate for automated referrals due to its high false‐positive rate. Considering resource implications that can result from falsely identifying patients as distressed when they are not, we suggest that the DT should not be used in isolation; rather, a follow‐up step is likely necessary to determine who requires additional support through multi‐item patient‐reported outcome measures or further discussion with clinicians to explore their areas of concern. Given the growing population of cancer survivors post‐oncologic surgeries, there is a pressing need to explore efficient tools for monitoring psychological burden, and this study nonetheless provides evidence supporting the DT's utility as a practical and valid distress screening tool. Given its clinical utility, future research should focus on validating the DT in oncologic surgical cohorts with complex surgeries across multiple centers to strengthen its broader clinical applicability.

### 4.2 Strengths and Limitations

Our current study has several strengths. This is the first validation study in a PE cohort, a group with known complex care needs, and our sample size (*n* = 377) is relatively large compared to other validation studies [15, 33, 43]. Additionally, this study contributes to the limited evidence on the responsiveness of the DT, providing support for using this tool to assess distress over time. Our study also examined clinical utility in determining an optimal cutoff score by assessing CUIs.

Our study also has some limitations. To assess the convergent validity and determine the optimal cutoff score of the DT, we used the EWB subscale as an external reference criterion, as it measures a construct related to distress. However, instruments such as the Hospital Anxiety and Depression Scale (HADS) and the Brief Symptom Inventory‐18 (BSI‐18) are more commonly used as reference measures, as they assess psychological constructs that are conceptually more closely aligned with distress, including anxiety, depression, and general psychological symptoms [45]. Additionally, the minimum increment of the DT scale was set at 0.5 in our electronic analogue scale, and a small number of cases (*n* = 8) were rounded up to the nearest whole‐number. While this did not alter the interpretation of the findings, whole‐number increments are commonly used when administering the DT, which one may argue limits the generalizability of our findings. Furthermore, this study was conducted at a single centre, which may restrict the wider applicability of the findings across different clinical settings. Participants included in the analyses also differed from those not included on some demographic and clinical characteristics, such as age, treatment intent, and exenteration type, which may limit the generalizability of the findings to the broader patient population. Finally, this study used data that was collected up to 16 years ago, and as clinical practice has changed over time, this may have influenced the distress levels. However, it could be argued that distress and perception of HRQL are universal phenomena, and given the study primarily examined associations between measures assessing these constructs, the findings are likely to remain valid.

## 5 Conclusion

This is the first study examining responsiveness and convergent validity of the DT in patients undergoing PE, reinforcing its utility as a brief and practical screening tool for psychological distress over time in this complex surgical population. Whilst the DT demonstrated good sensitivity, the high false‐positive rate suggests its use as a screening tool with follow‐up assessment for those who screen positive, rather than as a trigger for automated referral. Nonetheless, our findings support the DT's potential for ongoing monitoring of distress in PE patients, facilitating timely referrals for appropriate support and contributing to improved post‐treatment care.

## Author Contributions

**Nicholas Melander:** methodology, writing review and editing. **Claudia Rutherford:** conceptualization, methodology, supervision, writing review and editing. **Daniel Steffens:** conceptualization, methodology, supervision, writing review and editing. **Sascha Karunaratne:** project administration, data curation, conceptualization, writing review and editing. **Kate Alexander:** project administration, data curation, conceptualization, writing review and editing. **Cherry Koh:** conceptualization, supervision, writing review and editing. **Michael Solomon:** conceptualization, supervision, writing review and editing. **Bora Kim:** project administration, data curation, formal analysis, conceptualization, methodology, writing original draft.

## Funding

The authors have nothing to report.

## Ethics Statement

The study received ethical approval and governance authorization from the Royal Prince Alfred Hospital Human Research Ethics and Governance Office (X13‐0283 and X20‐0392 for the original conduct of the study, and X24‐0115 for secondary analysis). The study was performed in accordance with the ethical standards specified in the 1964 Declaration of Helsinki and its later amendments or comparable ethical standards.

## Consent

All participants provided written informed consent.

## Conflicts of Interest

The authors declare no conflicts of interest.

## Supporting information


Supporting Information S1

## Data Availability

The data that support the findings of this study are available on request from the corresponding author. The data are not publicly available due to privacy or ethical restrictions.
