# Supplementary material for: Evaluation of the Distress Thermometer in Pelvic Exenteration: Responsiveness‐To‐Change, Convergent Validity, and Clinical Cutoff
Source: Psychooncology. 2026 Aug 14;35(8):e70565. doi: 10.1002/pon.70565 (PMC13474108; doi:10.1002/pon.70565)
Supplement: Supplementary file 1 [file PON-35-e70565-s001.docx]

Supplementary 1. Frequency distribution of DT scores

A

| DT score | Baseline | |  | 6 month follow-up | |
| --- | --- | --- | --- | --- | --- |
|  | N | % |  | N | % |
| 0 | 49 | 13.0 |  | 98 | 26.0 |
| 1 | 47 | 12.5 |  | 50 | 13.3 |
| 2 | 36 | 9.5 |  | 62 | 16.4 |
| 3 | 30 | 8.0 |  | 33 | 8.8 |
| 4 | 21 | 5.6 |  | 29 | 7.7 |
| 5 | 56 | 14.9 |  | 39 | 10.3 |
| 6 | 39 | 10.3 |  | 15 | 4.0 |
| 7 | 33 | 8.8 |  | 21 | 5.6 |
| 8 | 34 | 9.0 |  | 15 | 4.0 |
| 9 | 12 | 3.2 |  | 6 | 1.6 |
| 10 | 20 | 5.3 |  | 9 | 2.4 |

B

Note: (A) Frequency table of DT scores; (B) Frequency graph of DT scores.

DT: distress thermometer.

Supplementary 2. ROC of DT scores versus the EWB subscale cutoff score.

A	B

Note. (A) ROC of DT scores versus the EWB subscale cutoff score at baseline, (B) ROC of DT scores versus the EWB subscale cutoff score at 6 months.

DT: distress thermometer; ROC: receiver operating characteristic; EWB: emotional wellbeing.

Supplementary 3. Frequency of DT scores grouped by the EWB cutoff.

A

B

Note. (A) Frequency of DT scores grouped by the EWB cutoff at baseline, (B) Frequency of DT scores grouped by the EWB cutoff at 6-months.

DT: distress thermometer; EWB; emotional well-being subscale.
